# Bayesian Correction for Misclassification in Multilevel Count Data Models

**DOI:** 10.1155/2018/3212351

**Published:** 2018-02-25

**Authors:** Tyler Nelson, Joon Jin Song, Yoo-Mi Chin, James D. Stamey

**Affiliations:** ^1^Department of Statistical Science, Baylor University, Waco, TX, USA; ^2^Department of Economics, Baylor University, Waco, TX, USA

## Abstract

Covariate misclassification is well known to yield biased estimates in single level regression models. The impact on hierarchical count models has been less studied. A fully Bayesian approach to modeling both the misclassified covariate and the hierarchical response is proposed. Models with a single diagnostic test and with multiple diagnostic tests are considered. Simulation studies show the ability of the proposed model to appropriately account for the misclassification by reducing bias and improving performance of interval estimators. A real data example further demonstrated the consequences of ignoring the misclassification. Ignoring misclassification yielded a model that indicated there was a significant, positive impact on the number of children of females who observed spousal abuse between their parents. When the misclassification was accounted for, the relationship switched to negative, but not significant. Ignoring misclassification in standard linear and generalized linear models is well known to lead to biased results. We provide an approach to extend misclassification modeling to the important area of hierarchical generalized linear models.

## 1. Introduction

Misclassification and measurement error is well known to cause bias in estimation. The exposure variable in epidemiologic studies is often subject to misclassification [1–3]. While the impact of misclassification is well documented for generalized linear models with a single level, considerable less work has been done on multilevel models. Covariate misclassification has been considered for continuous outcome models [4, 5]. Here, our focus is on multilevel models with a count response where the primary exposure of interest is potentially misclassified.

Partner violence can have impacts on a wide range of outcomes on the abused partner, for instance, depression and even suicide attempts [6, 7]. The impact on children in the household of domestic violence has also been studied [8–10]. Domestic violence is well known to be misclassified, especially in survey type data [11].

Our analysis uses India National Health Survey (NFHS-3) 2005-2006. It is a nationally representative household survey that provides a set of key variables for the study, including the number of surviving children a woman has and the occurrence of partner violence in the previous generation which is subject to misclassification. Thus our interest is in determining if previous generation spousal abuse impacts number of children correcting for potential misclassification in reported spousal abuse.

Our paper is organized as follows. We first describe the National Family and Health Survey. Next, we discuss the proposed Bayesian hierarchical model that accounts for covariate misclassification. We discuss the results of a simulation experiment in which we compare the proposed model to the naïve model that ignores misclassification. We apply the proposed model to the data from National Family and Health Survey and conclude with a discussion.

## 2. Data Description

According to the National Family and Health Survey in 2005, total lifetime prevalence of domestic violence was 33.5% and 8.5% for sexual violence among women aged 15–49. More notably, lifetime prevalence of domestic abuse ranged from 18% to 45% in states across India. In [12], it is explored whether female employment enhances women's bargaining power within the home and reduce the risk of intimate partner violence. Further, [13] examined whether female employment challenges the culture of male dominance and provokes more violence. Lastly, [14] used many of the factors from the previous studies but also addressed misclassification bias in violence reporting due to the significant underreporting of intimate partner violence in surveys [15]. We explore whether being raised in a household with spousal abuse significantly impacts the number of children the child exposed has in their lifetime. Data used in the analysis came from India National Health Survey (NFHS-3) 2005-2006. The sample is restricted to urban (mega city, large city, small city, and large town) women from 27 different states and key variables are physical spousal violence experience of their parents and the number of children they have. Additional covariates include a wide range of demographic and socioeconomic variables. The sample includes 19,026 urban women that have no missing variables. Lastly, we assume the spousal abuse rate varies across the 27 different states as well as the number of children.

## 3. Model

We follow the general approach of [16] for measurement error models. The approach is to specify three models, the outcome model, exposure model, and measurement model. The outcome model we consider has two levels with *i* = 1, 2,…, *n* denoting subjects and *j* = 1, 2, …, *N* denoting clusters. The count data at level 1 are assumed to be Poisson:

(1) $$\begin{array}{c} y_{ij}\sim Poisson\left(\;r_{ij}\lambda_{ij}\;\right), \end{array}$$

where *r*_*ij*_ is an offset, often time or space, and *λ*_*ij*_ is the rate of occurrence which is related to covariates through a log-linear model:

(2) $$\begin{array}{c} \log\left(\;\lambda_{ij}\;\right)=\beta_{0j}+\beta_{1}x_{ij}+\overset{p}{\underset{k=2}{\sum }}\beta_{k}z_{ijk}. \end{array}$$

The exposure, *x*_*ij*_, is assumed to be potentially misclassified. Other covariates (*z*_*ijk*_) are assumed to be measured without error. *β*_0*j*_ represents a random intercept for each cluster:

(3) $$\begin{array}{c} \beta_{0j}=\beta_{0}+u_{j} \end{array}$$

with *u*_*j*_ ~ *N*(0, *σ*^2^). *β*_1_ is the exposure effect of primary interest while *β*_*k*_, *k* > 1, represents the impact of other covariates. The exposure model for binary covariate *x*_*ij*_ is assumed to be a logistic model:

(4) $$\begin{array}{c} x_{ij}\sim Bernoulli\left(\;\pi_{ij}\;\right) \end{array}$$

with

(5) $$\begin{array}{c} logit\left(\;\pi_{ij}\;\right)=\gamma_{0j}+\overset{q}{\underset{k=1}{\sum }}\gamma_{k}z_{ijk} \end{array}$$

and random intercept

(6) $$\begin{array}{c} \gamma_{0j}=\gamma_{0}+v_{j} \end{array}$$

with *v*_*j*_ ~ *N*(0, *ν*^2^) and *γ*_0*j*_. Finally, the measurement model depends on several assumptions and the amount of data available. Here, we assume nondifferential misclassification and either one or two tests available used as surrogates for the true exposure, *x*_*ij*_. In the one test case, we assume the fallible method has sensitivity *S*_1_ and specificity *C*_1_ and conditional on the true value we have fallible assessment *x*_1*ij*_ which takes values of 0 and 1 with probability

(7) fx1ij ∣ xij=S1x1ij1−S11−x1ijxij×C11−x1ij1−C1x1ij1−xij.

If there are two conditionally independent assessments, then the measurement model is in terms of two fallible outcomes *x*_1*ij*_ and *x*_2*ij*_ with sensitivities *S*_1_ and *S*_2_ and specificities *C*_1_ and *C*_2_ and probability function:

(8) fx1ij ∣ xij=S1x1ij1−S11−x1ijxij×C11−x1ij1−C1x1ij1−xij×S2x2ij1−S21−x2ijxij×C21−x2ij1−C2x2ij1−xij.

**Figure 1:**
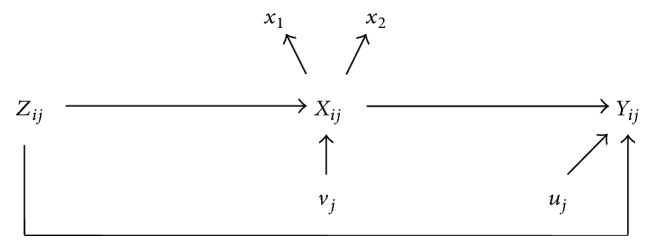
Directed acyclic graph illustrating how the outcome model, exposure model, and measurement model are connected. *x*_1_ and *x*_2_ are the fallible assessments of the true exposure, *X*_*ij*_. The covariates measured without error, *Z*_*ij*_, describe both *X*_*ij*_ and the outcome *Y*_*ij*_. Lastly, the random effects for both the exposure model and the outcome model are represented by *v*_*j*_ and *u*_*j*_, respectively.

Figure 1 provides a graph showing the relationship between all the variables in the model.

Because we are using the Bayesian framework we require prior distributions for the model parameters. In the absence of prior information or expert opinion, diffuse normal prior distributions are often used for logistic and Poisson regression coefficients; see, for example, [17, 18]. Thus, we assume that *γ*_0_, *γ*_1_,…, *γ*_*q*_ have independent normal prior distributions with mean 0 and variance 10. Similarly, the Poisson regression coefficients, *β*_0_, *β*_1_,…, *β*_*p*_, are also assumed to be independent and normally distributed but we assume a variance of 100 for these. We assume uniform priors for the random effects standard deviations *σ* and *ν* [19]. Finally, beta priors are used for the sensitivities and specificities. For the two assessment case, noninformative beta (1, 1) priors can be used. If only one assessment is used, the model is not identifiable and thus requires informative priors for both *S*_1_ and *C*_1_.

The joint posterior is proportional to the product of the likelihoods from the outcome, exposure, and measurement models along with the prior distributions. The resulting marginal posteriors for all parameters of interest are not available in closed form. Packages such as OpenBUGS and JAGS can be used to fit the model. Our OpenBUGS code is available from the first author upon request.

## 4. A Simulation Study

To determine the size of the impact of the covariate misclassification on the hierarchical count model we performed a simulation experiment. We generate the simulated data in order to mimic the real data. Specifically, we assume a single exposure variable that is subject to misclassification along with four other covariates that are assumed to be measured correctly. The true exposure model is a binomial model with a logit link:

(9) logitπij=−1.5+νj−0.21zij1−0.16zij2+0.19zij3−0.29zij4

while the Poisson log-linear model for the outcome is

(10) logλij=0.85+uj+0.4xij+0.25zij1−0.2zij2−0.18zij3−0.05zij4,

where *ν*_*j*_ ~ *N*(0,0.1^2^) and *u*_*j*_ ~ *N*(0,0.1^2^). These random effect standard deviations of 0.1 are relatively small. We also considered the cases of 0.2 and 0.3 and found the results to be similar. For the measurement model we consider two cases. The first is with a single diagnostic test with sensitivity *S*_1_ and specificity *C*_1_. As a second case, we assume two conditionally independent tests, the first with sensitivity *S*_1_ and specificity *C*_1_ and the second with sensitivity *S*_2_ and specificity *C*_2_. For the first test we fix *S*_1_ = 0.55 and *C*_1_ = 0.95. For the second test we have *S*_2_ = 0.7 and *C*_2_ = 0.8.

For the simulation we set* N*, the number of clusters, to be 25 and *n*_*i*_ = 200 for all clusters. We simulated 50 data sets and fit three models. The first is the naïve model where the misclassified covariate is assumed to be measured without error. The second model is the case of a single fallible diagnostic test. Finally, we consider the case of two conditionally independent diagnostic tests. For the case of a single diagnostic test we require informative priors for the sensitivity and specificity. We use a beta (10, 8) for *S*_1_ and a beta (165.7, 9.7) for *C*_1_. Note the prior mean for *S*_1_ is 0.55 and the prior mean for *C*_1_ is 0.94. So the priors are centered near the truth, but the 95% prior interval for *S*_1_ is (0.33, 0.77), which allows for a wide range of possible values. These priors are consistent with [15] which accounts for errors in the domestic abuse question in a survey. Note there is considerably more certainty about the specificity than the sensitivity. This makes intuitive sense in that people who are not victims are unlikely to say they are, but victims are much more likely to underreport. For the rest of the parameters we use noninformative priors, specifically, normal (0, 10) for the logistic regression coefficients, normal (0, 100) for Poisson coefficients, and uniform (0, 3) priors for both random effect standard deviations. For the two diagnostic test cases, we do not require informative priors, and use beta (1, 1) priors for *S*_1_, *C*_1_, *S*_2_, and *C*_2_.

The results across the 50 simulations for the main parameter of interest, *β*_1_, are displayed Table 1. Note the true value is *β*_1_ = 0.4 The naïve model performs particularly poorly illustrating the problem of ignoring the misclassification. The average posterior mean across the 50 data sets is approximately 0 and none of the 95% intervals contained the true value. The two corrected models performed significantly better with both having empirical coverage over 90% and significantly less bias. The main cost of the misclassification model over the naïve is larger variability. For instance, the posterior standard deviation for *β*_1_ averages only 0.025 for the naïve model but is about 0.11 for both misclassification models. Accounting for misclassification and measurement error commonly inflates variability in estimates, but this is preferred to the large bias and low coverage of the naïve model. Though *β*_1_ has the largest degree of bias for the naïve model, most of the other parameters are also somewhat biased and coverages of the intervals below nominal. The performance for all the parameters are provided in Appendix.

## 5. Application

We apply the proposed Poisson regression model with a misclassified covariate to the India National Health Survey (NFHS-3) 2005-2006 data accounting for the 27 states as a cluster random effect. We consider five covariates: a binary indicator for whether a parent reported being abused, the age of the female, the education level of the female, a binary indicator as to whether the family considers themselves religious, and a continuous variable indicating their wealth. The number of children each woman surveyed has is the outcome variable and we assume the counts are distributed Poisson. All of the variables are considered to be reported correctly other than the binary variable indicating whether a female was abused or not. Because the one test Poisson model is nonidentifiable we must supply the prior on the sensitivity and specificity relatively informative beta distributions. Information from [14, 15, 19] on reporting of partner violence in developing countries indicates the distribution of the sensitivity could easily vary between 20 and 60%. Using the same sources, the specificity is found most likely to be above 90%. Using this information, we specify the priors as* S* ~ Beta (5, 7.6) and* C* ~ Beta (165.7, 9.7). The priors indicate a substantial degree of underreporting with a prior mean of only 40% for the sensitivity. Conversely, the specificity is expected to be quite high, with a prior mean over 94%. We also consider the naïve model and report the posterior estimates of the response model; however, it is unnecessary to consider the exposure model.

We fit the data with both the proposed model and a naïve model that does not account for the misclassification. The models were fit in OpenBUGS and inferences were based on 20,000 iterations after 10,000 burn-ins. The results for both models are provided in Table 2. The main parameter of interest is the spousal abuse parameter, *β*_1_. For the naïve model, the posterior of *β*_1_ is positive and the 95% interval is completely above 0 indicating statistical significance. Thus the inference would be that parental partner violence would lead to an increase in number of children. For the misclassification model, the coefficient is not significant as the interval does contain 0. This is not unusual because misclassification is well known to bias estimates towards the null. What is particularly interesting here is that though the interval contains 0, the posterior mean is negative, thus by accounting for the misclassification the majority of the posterior shifted from positive to negative. This further illustrates the importance of accounting for covariate misclassification in hierarchical count models. The other covariates in the response model are all significant and are very similar in both models. Age has a positive relationship with number of children while education, religiosity, and wealth all have negative relationships. The naïve model has no estimates for the *γ* parameters or the sensitivity and specificity since these are all concerned with the misclassification model. For the misclassification model, we see that age, education, and wealth all reduce the probability of abuse among the parents and are statistically significant. Religiosity appears to have a positive relationship with abuse, but the 95% interval contains 0, so the relationship is not strong. The standard deviation of the random slopes for the outcome model is relatively modest with a posterior mean of 0.13. This indicates only a small degree of heterogeneity of the baseline average number of children across the 27 states. For the misclassification model, the random effect standard deviation has a posterior mean of 1.12 indicating considerably more heterogeneity of the baseline probability of abuse across the 27 states. Finally, the sensitivity and specificity both have posteriors that are centered around larger values than the priors. This indicates there is less misclassification in the data than was expected, but still considerable underreporting since the sensitivity is still centered around a value less than 0.5.

## 6. Conclusion

In this paper, we have addressed the problem of covariate misclassification in a hierarchical count model. Through simulation, we illustrated that ignoring the misclassification can lead to biased estimates and undercoverage of interval estimators. Our real data example demonstrated an extreme possibility in that the naïve model yielded results that were statistically significant (95% interval completely above 0) while the bias corrected model had a point estimate that was negative, though not statistically significant. There are several extensions to the model we have proposed. In some cases, the count response may also be subject to misclassification. Accounting for under- or overreporting of the response in a hierarchical model such as this would be an interesting follow-up work. An important limitation to note is that while the information for our priors for the example is from developing countries including studies from India specifically, we have not matched subjects with our current data; thus there is no guarantee that the populations match perfectly. This is one reason why the two diagnostic case approaches are preferred because the information on the sensitivity and specificity come from the current data. However, the one diagnostic test case essentially works as a Monte Carlo sensitivity analysis where the priors dictate a range of likely values for the sensitivity and specificity. In that sense, our results are robust with respect to a large number of possible true values of the sensitivity and specificity.

## Figures and Tables

**Table 1:** Averages across 50 simulated data sets for *β*_1_. True value is 0.4.

	Mean	SD	Coverage
Naïve	0.001	0.03	0
One test	0.38	0.11	1
Two tests	0.34	0.11	0.91

**Table 2:** Posterior summaries for naïve and misclassification models.

		Mean	SD	95% interval
Naïve results	*β*_0_	1.02	0.03	(0.96, 1.08)
	*β*_1_	0.03	0.01	(0.01, 0.06)
	*β*_2_	0.25	0.01	(0.24, 0.26)
	*β*_3_	−0.19	0.01	(−0.20, − 0.17)
	*β*_4_	−0.19	0.01	(−0.21, − 0.17)
	*β*_5_	−0.07	0.01	(−0.08, − 0.05)
	*σ*	0.13	0.02	(0.09, 0.18)

Assuming misclassification	*β*_0_	1.03	0.03	(0.96, 1.09)
	*β*_1_	−0.02	0.02	(−0.05, 0.02)
	*β*_2_	0.25	0.01	(0.24, 0.26)
	*β*_3_	−0.19	0.01	(−0.20, − 0.18)
	*β*_4_	−0.19	0.01	(−0.21, − 0.17)
	*β*_5_	−0.07	0.01	(−0.08, − 0.06)
	*σ*	0.13	0.02	(0.09, 0.18)
	*γ*_0_	−1.78	0.26	(−2.27, − 1.25)
	*γ*_1_	−0.19	0.04	(−0.26, − 0.12)
	*γ*_2_	−0.5	0.06	(−0.62, − 0.39)
	*γ*_3_	0.14	0.08	(−0.01, 0.29)
	*γ*_4_	−0.44	0.06	(−0.56, − 0.34)
	*ν*	1.12	0.21	(0.78, 1.62)
	*C*	0.98	0.01	(0.97, 0.99)
	*S*	0.52	0.05	(0.44, 0.61)

**Table 3:** Simulation results for naïve model.

	Truth	Mean	SD	Coverage
*β*_0_	0.85	0.94	0.02	0.04
*β*_1_	0.4	0.00	0.03	0
*β*_2_	0.25	0.24	0.01	0.66
*β*_3_	−0.2	−0.21	0.01	0.78
*β*_4_	−0.18	−0.17	0.01	0.73
*β*_5_	−0.05	−0.07	0.01	0.39
*σ*	0.1	0.10	0.02	0.96

**Table 4:** Simulation results for one diagnostic test case.

	Truth	Mean	SD	Coverage
*β*_0_	0.85	0.86	0.04	0.87
*β*_1_	0.4	0.37	0.11	1
*β*_2_	0.25	0.25	0.01	0.98
*β*_3_	−0.2	−0.2	0.01	0.93
*β*_4_	−0.18	−0.18	0.01	0.96
*β*_5_	−0.05	−0.04	0.01	0.97
*γ*_0_	−1.5	−1.76	0.40	0.94
*γ*_1_	−0.21	−0.3	0.11	1
*γ*_2_	−0.16	−0.21	0.1	0.96
*γ*_3_	0.19	0.28	0.11	0.9
*γ*_4_	−0.29	−0.39	0.12	0.96
*S*	0.55	0.52	0.09	0.94
*C*	0.95	0.93	0.02	0.88
*σ*	0.1	0.19	0.13	0.98
*ν*	0.1	0.1	0.02	0.94

**Table 5:** Simulation results for two diagnostic test cases.

	Truth	Mean	SD	Coverage
*β*_0_	0.85	0.86	0.04	0.94
*β*_1_	0.40	0.34	0.11	0.91
*β*_2_	0.25	0.24	0.01	0.94
*β*_3_	−0.20	−0.20	0.01	0.95
*β*_4_	−0.18	−0.17	0.01	0.98
*β*_5_	−0.05	−0.05	0.01	0.92
*γ*_0_	−1.50	−1.61	0.25	0.92
*γ*_1_	−0.21	−0.21	0.06	0.96
*γ*_2_	−0.16	−0.16	0.06	0.98
*γ*_3_	0.19	0.18	0.06	0.92
*γ*_4_	−0.29	−0.29	0.06	0.96
*S*_1_	0.70	0.57	0.07	0.95
*S*_2_	0.55	0.72	0.07	0.94
*C*_1_	0.95	0.94	0.01	0.90
*C*_2_	0.90	0.89	0.02	0.92
*τ*_*a*_	0.10	0.12	0.08	1.00
*τ*_*c*_	0.10	0.10	0.02	0.98

## Appendix

In Tables 3–5 we provide the summaries for all parameters from the simulation study of Section 2.
